# Impact on Clinical- and Patient-Reported Outcomes Measures of an Organ Preservation-Based Therapeutic Strategy in Locally Advanced Rectal Cancer: The FOREST Project

**DOI:** 10.3390/jcm15020844

**Published:** 2026-01-20

**Authors:** Hector Guadalajara, Ion Cristóbal, Raquel Fuentes-Mateos, Eva Ruiz-Hispán, Jose Luis Domínguez-Tristancho, Miguel Leon-Arellano, Paula Sánchez-Moreno, Marta Sabater-Durán, Juan Antonio Álvaro de la Parra, Damián García-Olmo, Cristina Caramés

**Affiliations:** 1 Department of General and Digestive Surgery, Fundación Jiménez Díaz University Hospital, 28040 Madrid, Spain; h.guadalajara@quironsalud.es (H.G.); jluis.dominguezt@quironsalud.es (J.L.D.-T.); miguel.leon@quironsalud.es (M.L.-A.); damian.garcia@quironsalud.es (D.G.-O.); 2 Department of Surgery, School of Medicine, Autonomous University of Madrid, 28049 Madrid, Spain; 3 Corporate Department of Healthcare and Research, Quirónsalud Healthcare Network, 28043 Madrid, Spain; psmo.edu@quironsalud.es; 4 Medical Oncology Department, Fundación Jiménez Díaz University Hospital, 28040 Madrid, Spain; raquel.fmateos@quironsalud.es (R.F.-M.); eva.ruizh@quironsalud.es (E.R.-H.); 5 Project Implementation and Processes Department, Quirónsalud Healthcare Network, 28043 Madrid, Spain; marta.sabaterd@quironsalud.es; 6 General Operations Directorate, Quirónsalud Healthcare Network, 28043 Madrid, Spain; jaalvaro@quironsalud.es; 7 Doctoral School of the UAM, Center for Postgraduate Studies, Autonomous University of Madrid, 28049 Madrid, Spain; 8 Doctoral Program in Medicine and Surgery, Autonomous University of Madrid, 28049 Madrid, Spain

**Keywords:** shared decision-making, organ preservation, patient-reported outcome measures, patient-reported experience measures, rectal cancer

## Abstract

**Background**: Locally advanced rectal cancer is traditionally managed with neoadjuvant chemoradiotherapy followed by total mesorectal excision, but radical surgery entails substantial morbidity, including bowel, urinary, and sexual dysfunction as well as permanent stomas. Organ-preserving strategies such as total neoadjuvant therapy (TNT) followed by a watch-and-wait (WW) approach aim to reduce morbidity while maintaining oncologic safety. A recent study from the FOREST cohort confirmed favorable survival outcomes with WW but did not assess the patient-centered impact. **Methods**: This retrospective observational study included locally advanced rectal cancer patients treated at a tertiary hospital. Following TNT, patients who achieved a complete clinical response entered WW, while others underwent radical surgery (RS). Patient-reported outcomes were assessed using an 18-item questionnaire grouped into domains and transformed to a 0–100 scale according to EORTC scoring methodology. All patients underwent a shared decision-making process. Comparisons between groups used Pearson chi-square tests for clinical and demographics associations and Mann–Whitney U tests for ordinal outcomes. The protocol was integrated into Quirónsalud’s value-based healthcare framework. **Results**: Clinical and demographics characteristics did not differ between WW and RS groups. PROMs favored WW in multiple domains: Symptoms/Complications (87 vs. 66; *p* < 0.001), Psychosocial adaptation (90 vs. 66; *p* < 0.001), Mental health (90 vs. 78; *p* = 0.006), and Global quality of life (80 vs. 67; *p* = 0.011). Bowel and sexual functions were similar between groups, and Care satisfaction was very high for both. **Conclusions**: TNT plus WW appears to be oncologically safe and confers significant quality-of-life benefits across several domains. These findings support the theory that WW is a value-based, patient-centered strategy for rectal cancer, and this warrants validation in larger, randomized cohorts.

## 1. Introduction

Colorectal cancer (CRC) remains a major global health challenge. According to GLOBOCAN 2022, CRC accounted for approximately 1.9 million new cases and 940,000 deaths worldwide, ranking third in incidence and second in cancer-related mortality [1]. Rectal cancer comprises about one-third of all CRC cases, with an estimated 730,000 new diagnoses and 340,000 deaths worldwide reported in 2022. Locally advanced rectal cancer (LARC), generally defined as stage II-III disease, requires multimodal treatment. The standard approach, neoadjuvant chemoradiotherapy followed by total mesorectal excision (TME), has significantly reduced local recurrence rates and improved survival [2,3]. However, radical surgery is associated with considerable long-term morbidity, including bowel dysfunction, sexual and urinary disturbances, and permanent stomas, all of which can severely impact quality of life [4].

In response to these limitations, organ preservation strategies have emerged as an alternative in recent years. Total neoadjuvant therapy (TNT), which administers systemic chemotherapy and radiation before surgery, has demonstrated improved pathological complete response (pCR) rates and a reduction in distant metastases [5,6,7]. This has allowed for the adoption of non-surgical management in selected patients. The “watch-and-wait” (WW) approach [8] involves postponing surgery in patients who achieve a complete clinical response (CCR) after neoadjuvant therapy, with close monitoring and salvage surgery in case of tumor recurrence. Multiple studies have validated this therapeutic strategy as oncologically safe, with overall survival (OS) and 5-year disease-specific survival (DFS) rates comparable to those of patients undergoing TME [9,10]. While WW carries a higher risk of local recurrence (approximately 20–25%), most cases are recoverable without compromising long-term outcomes [11]. Beyond oncological safety, the WW approach offers functional advantages. Patients who avoid surgery report improved bowel, urinary, and sexual function, as well as better quality of life [5]. Moreover, recent randomized data from the OPRA trial further support the feasibility of organ preservation in LARC, observing that approximately 50% of patients could avoid surgery through a WW strategy, with comparable DFS across arms [12]. These findings reinforce the role of TNT in increasing complete response rates and enabling non-operative management, while maintaining oncologic safety. However, active surveillance also carries psychological burdens, particularly anxiety related to recurrence and the intensity of follow-up. These aspects are often underrepresented in clinical assessments, highlighting the need for patient-centered outcome indicators.

This is where patient-reported outcome measures (PROMs) and patient-reported experience measures (PREMs) become essential. PROMs collect patients’ assessments of their symptoms, function, and quality of life, while PREMs reflect their experiences with the care they receive. Their integration in oncology has demonstrated benefits in communication, symptom management, and even survival [13,14]. In rectal cancer, PROMs are critical for assessing functional outcomes after treatment. However, existing instruments (e.g., EORTC QLQ-C30/CR38) were developed for surgical populations and may not adequately reflect the experience of patients who have not undergone surgery [15]. A qualitative study by Pennings et al. [16] revealed that patients following a WW protocol face persistent symptoms and emotional distress, underscoring the need for tailored PROMs and supportive care.

The FOREST project was conceived as a comprehensive therapeutic management protocol for LARC with elective neoadjuvant therapy. Its design aimed to address the current existing gaps by integrating oncologic endpoints with PROMs and PREMs to assess treatment value from both clinical and patient perspectives, comparing outcomes from radical surgery and WW following TNT in LARC patients. In a previous work, we evaluated both treatment groups to determine whether WW can offer equivalent oncological safety with superior patient-centered value. Notably, the FOREST protocol demonstrated that integrating TNT with response-guided strategies enables high organ preservation rates in rectal cancer without compromising oncologic safety. Patients managed with a WW approach showed comparable disease-free survival and significantly better overall survival than those undergoing radical surgery [17]. The FOREST protocol is grounded in a patient-centered philosophy, where shared decision-making plays a central role and has been reported to provide clinical benefits [18]. Treatment pathways are not solely determined by clinical response but are co-designed with patients, respecting their values, preferences, and tolerance for risk.

## 2. Materials and Methods

### 2.1. Study Design and Objectives

The FOREST study (Facilitating Oncological Results and Experience for Sustainable Treatment) is a prospective observational cohort designed to evaluate clinical and patient-centered outcomes in the management of locally advanced rectal cancer (LARC). The primary objective of the study was to evaluate patient-reported outcomes and experiences in those patients enrolled in a FOREST project based on organ preservation without compromising oncologic safety. The study included all available data from LARC patients included in the FOREST project whose endoscopic biopsy was performed between October 2020 and December 2022.

### 2.2. Study Population and Eligibility Criteria

**Figure 1:**
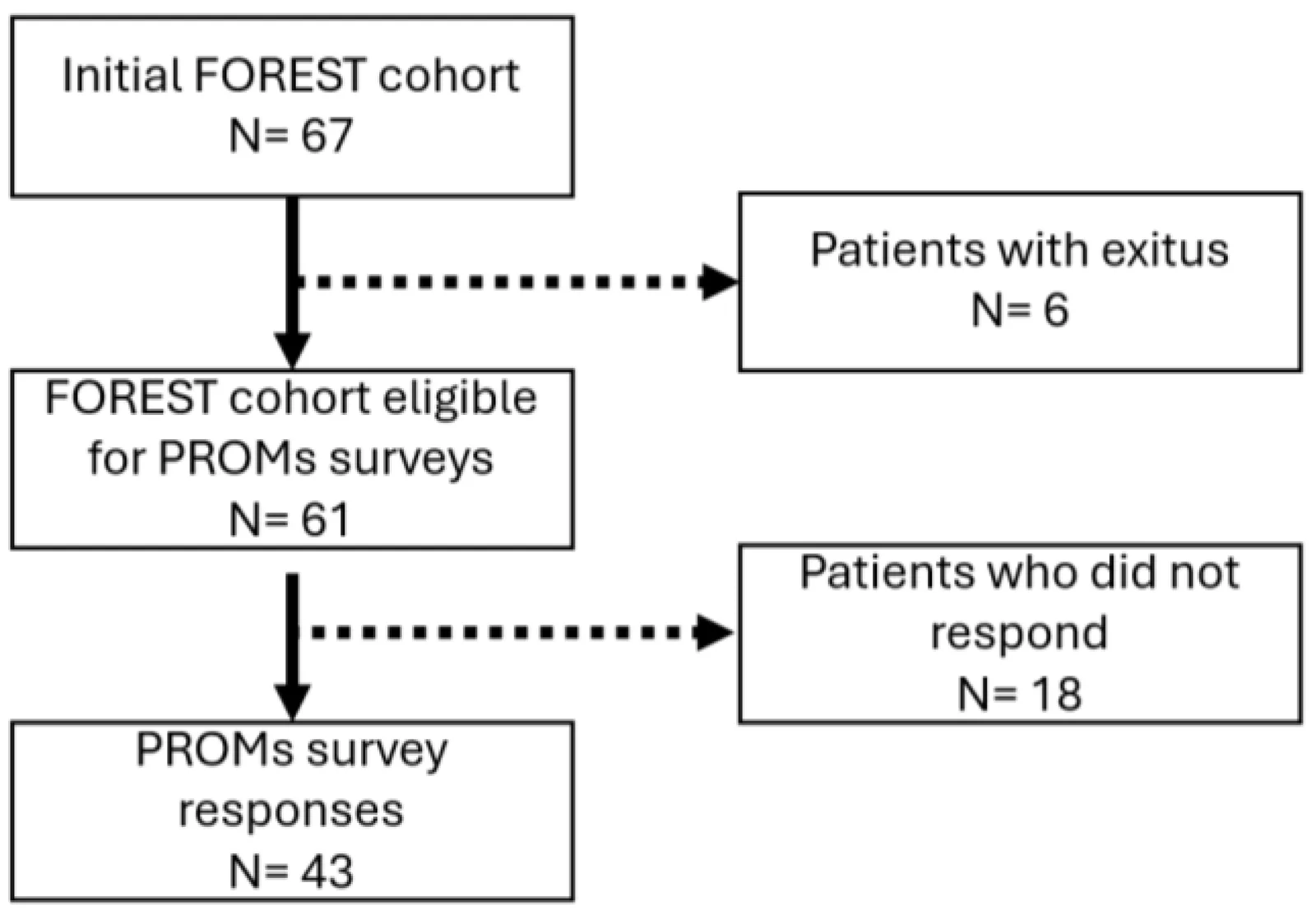
Flowchart of patients who responded to the PROMs survey and were included in the study. Solid arrows indicate patients who remain in the study, and dotted arrows indicate patients who left the study.

A total of 67 patients diagnosed with LARC at Fundación Jiménez Díaz University Hospital and included in the FOREST cohort were initially considered for this study. The FOREST study’s inclusion criteria were as follows: a histologically confirmed rectal adenocarcinoma, and cases who met the following clinical staging criteria: cT1N1, or cT2–T4 with any N stage, M0, and microsatellite stability (MSS). All patients underwent baseline staging with pelvic MRI, endoscopy, and thoracoabdominal CT. Multidisciplinary tumor board review was performed prior to inclusion. The study excluded 6 patients who died and 18 cases who did not respond to the PROMs survey, resulting in a final number of 43 patients (Figure 1).

### 2.3. Clinical Protocol and Evaluation Schedule

All patients underwent an initial long-course chemoradiotherapy (CRT) regimen consisting of 54 Gy in 30 fractions and concomitant chemotherapy via capecitabine 825 mg/m^2^ given orally twice daily; this was taken continuously throughout the radiotherapy period. Following CRT, consolidation chemotherapy was administered starting on day 15 post-radiotherapy, with two cycles of FOLFOX in an approach consistent with TNT. FOLFOX-based chemotherapy (folinic acid, 5-fluorouracil, and oxaliplatin) was delivered every 15 days for a total of 6–8 cycles [17]. An early response assessment was performed at weeks 4–6 after completion of RT. Patients with a clinical complete response (cCR) at early evaluation were managed with a WW strategy, and those cases with an incomplete response continued with consolidation chemotherapy. Patients were stratified by risk in low-risk (cT2, T3ab, N0–1) receiving 6 cycles of FOLFOX, or high-risk (cT3cd, T4, any N) receiving 8 cycles of FOLFOX. Response reassessments and treatment decisions were informed by evaluations conducted at weeks 10–12 and 18–20 post-radiotherapy. Notably, patient stratification was based on the reassessed post-treatment clinical stage, not the initial stage. A late evaluation was conducted at weeks 24–26 after ending RT: Patients with cCR continued WW; patients with incomplete response underwent total mesorectal excision (TME); patients with near-complete response or those with tumor regrowth during WW were considered for local excision. All treatment decisions were made within a multidisciplinary framework, incorporating shared decision-making and patient preferences.

### 2.4. Patient-Reported Outcomes (PROMs)

PROMs were assessed using an 18-item ordinal questionnaire. Responses were coded as integers in ascending order, from the worst to the best outcome (lowest category = 1, next = 2, etc.), following the conventions of validated general cancer QoL instruments such as the EORTC QLQ-C30 with the colorectal-specific CR29 module, as well as condition-specific scales like the Low Anterior Resection Syndrome (LARS) score for bowel function and Vaizey incontinence score [15,19]. Missing responses were excluded (no imputation). The coding scheme was reviewed by at least two different clinicians to ensure that it reflected the intended hierarchy of outcomes. The follow-up time of the PROMs cohort was calculated from the date of the diagnostic biopsy to the date of PROM collection/last follow-up recorded in the PROMs dataset.

PROMs were collected after a minimum follow-up of two years from the initial diagnosis in all patients. Due to the real-world nature of the study, assessments were not performed at an identical time point for every individual. At the time the study was initiated, systematic prospective collection of PROMs was not yet implemented at our institution, and therefore a strictly standardized assessment schedule was not feasible. Thus, PROMs were collected once, as a cross-sectional long-term assessment, at the latest available follow-up recorded in the PROMs dataset.

### 2.5. Value-Based Healthcare Integration

The FOREST protocol is integrated into Quirónsalud’s VBHC strategic framework, which aims to optimize the outcomes that matter most to patients. The study incorporates digital tools for remote monitoring and patient participation, reflecting a care model that transcends traditional hospital boundaries. Final treatment decisions were made through a structured shared decision-making process involving the multidisciplinary tumor board and the patient. In cases of partial or incomplete clinical response, patients were fully informed of the risks and benefits of each option and could opt for a WW strategy even when radical surgery was clinically recommended. This approach allows for the professionalization of doctor–patient communication and deliberation in decision-making based on best practices, incorporating the values and preferences of the patient.

### 2.6. Statistical Analyses

Demographic and baseline characteristics were summarized by treatment arm using descriptive statistics: categorical variables as frequencies and percentages; continuous and ordinal variables as medians and interquartile ranges and as means with standard deviations where appropriate. We applied the Chi-2 test (Fisher exact test) based on the bimodal distribution of data to analyze the correlation final therapeutic plan and the clinical and demographic variables. Statistical analyses were performed using SPSS 27 for windows (SPSS Inc., Chicago, IL, USA). *p* < 0.05 was considered statistically significant.

The primary comparisons between the WW and RS cohorts were based on PROMs. Each questionnaire item was ordinal, and responses were mapped to integers in ascending order (lowest category = 1, next = 2, etc.), following conventions of validated instruments such as the EORTC QLQ-C30 and colorectal modules [15,19]. Missing or invalid responses were excluded listwise, and no imputation was performed. Items were grouped into clinically meaningful domains consistent with prior literature [5]. Symptoms and complications (items on presenting symptoms, pain, treatment sequelae, fatigue, neuropathy), Bowel function (items on sphincter control, urgency, ostomy-related issues), Sexual function (sexual life), Psychosocial adaptation (social life, work situation, family life), Psychological well-being (anxiety/worry, perception of cure), Care satisfaction (medical team, nursing team, information provided), and Global quality of life (overall QoL score). The domain-level analysis has been calculated using the EORTC linear transformation to a 0–100 scale. Comparisons were performed using the Mann–Whitney U test for independent samples (two-sided, α = 0.05). For each comparison, we report n per group, mean, and *p*-value. This approach aligns with standard practice in rectal cancer QoL research and recommendations for nonparametric analysis of PROMs [20].

### 2.7. Ethics and Reporting Standards

The study was conducted in accordance with the ethical standards of the institutional and national research committees and the Declaration of Helsinki, and the study protocol was reviewed and approved by the Research Ethics Committee of the Fundación Jiménez Díaz University Hospital (ref. EO157-20). Written informed consent was obtained from all participants. Data confidentiality and patient privacy strictly maintained, with all collected information anonymized and stored securely in compliance with applicable data protection regulations. STROBE reporting guidelines were followed in the preparation of this manuscript.

## 3. Results

### 3.1. Clinical and Demographic Characteristics

The final LARC patient cohort included 43 cases, with a mean age of 63.9 years (range: 33–85) and a sex distribution of 65% male and 35% female. The baseline characteristics of the study population are summarized in Table S1. We analyzed the association of the final therapeutic plan (WW or RS) with clinical and demographic characteristics to identify any baseline factors that might bias PROM responses (Table 1).

As expected, gender, age and histological subtype were not significantly associated with the likelihood of WW or RS. Notably, late morbidity rates were slightly higher in RS patients (19% in WW vs. 35.3% in RS), but without reaching significance (*p* = 0.258). Similarly, the prevalence of local and/or distant relapse and the proportion of cases living with disease were comparable between the WW and RS groups.

### 3.2. Clinical Evolution and Outcomes

We explore the data reported in the early evaluation (week 4–6), observing that all cases were able to proceed with TNT, except one that underwent immediate surgery due to a lack of response at that early time point. Notably, all cases that achieved an initial complete response at the early evaluation ultimately followed a WW strategy. Moreover, a similar proportion of cases who were treated with standard or extended TNT regimens were linked to a WW or RS strategy. Notably, all 16 patients who achieved complete response in the clinical late evaluation (week 20–26) were managed following a WW strategy, and 91.7% of cases who showed an incomplete response were treated with a RS therapeutic approach (Table 2).

### 3.3. Evaluation of PROMs Questionnaires

A total number of 43 LARC patients from the FOREST cohort completed the PROMs questionnaire and were eligible for this evaluation (25 in the Watch and Wait subgroup and 18 in the Radical Surgery one). The median follow-up for these 43 patients was 1295 days (IQR 1003–1484; range 851–2353). The follow-up duration was similar between the two strategy groups: in the WW cohort (*n* = 23), the median follow-up was 1295 days (IQR 978–1409; range 851–2353), and in the RS cohort (*n* = 20) it was 1308 days (IQR 1043–1545; range 894–1677).

The questionnaire comprised 18 ordinal items designed to capture patient-reported outcomes across multiple dimensions of recovery and wellbeing. Each item was coded numerically for statistical analysis following the scheme detailed in Table S2, ensuring that higher scores consistently reflected better outcomes. Table S2 provides the text of each question), the possible responses, and the numeric value assigned to each response during analysis).

To facilitate interpretation, individual items were grouped into clinically significant domains as defined in Table S3: Symptoms and complications (items 1, 2, 3, 8, 9), Bowel function and control (items 4, 5), Ostomy-related control (item 6), Sexual function (item 7), Psychosocial adaptation (items 10, 11, 12), Mental health (items 13, 14), Care satisfaction (items 15, 16, 17), and Global quality of life (item 18).

### 3.4. Domain-Level Analysis

The Symptoms and complications domain showed a clear advantage for WW patients, with a mean composite score of 87.5 vs. 62.5 (*p* < 0.001) (Table 3). This domain aggregates perceptions of symptom resolution, pain, complications, fatigue, and neuropathy, all of which were more favorable with a WW-based therapeutic plan. These findings suggest that organ-preserving management is associated with better control of treatment-related sequelae and improved physical comfort.

Bowel function and control, including sphincter control and defecatory urgency, showed similar average scores between the WW and RS groups, indicating that both strategies present comparable functional challenges for patients in this domain. Ostomy-related control also showed no significant differences; however, it should be noted that nearly all patients with a stoma were in the RS group. Sexual function scores were higher in the WW group than in the RS group (59.7 vs. 37.5), although this difference did not reach statistical significance; (*p* = 0.066).

In contrast, Psychosocial adaptation was markedly better in WW patients (mean 90.4 vs. 65.7; *p* < 0.001), reflecting easier reintegration into social life, work, and family roles after treatment. Similarly, Mental health scores were significantly higher in WW (90.0 vs. 77.8; *p* = 0.006), suggesting reduced anxiety and a stronger perception of recovery in this group. Care satisfaction was high in both cohorts, with only a modest, non-significant increase in the WW group (93.3 vs. 86.4; *p* = 0.105). Finally, Global quality of life was significantly better in the WW group (80.1 vs. 67.3; *p* = 0.011), consistent with the overall trend favoring organ preservation.

### 3.5. Item-Level Comparisons

Next, we analyzed each questionnaire item individually to provide more granular insights (Table 4). In the Symptoms and complications domain, WW patients consistently outperformed RS ones across all five items. Symptom resolution (item 1) averaged 3.92 vs. 3.56 (*p* = 0.006), pain control (item 2) 3.88 vs. 3.44 (*p* = 0.024), and fatigue (item 8) 3.92 vs. 3.28 (*p* < 0.001). Neuropathy (item 9) showed one of the largest gaps (3.52 vs. 2.53; *p* < 0.001). Finally, WW patients perceived lower levels of complications or treatment sequelae (item 3), although this difference was of borderline significance (2.88 vs. 2.28; *p* = 0.056).

Within the Bowel function and control domain, neither sphincter control (item 4: 3.18 vs. 3.10; *p* = 0.98) nor urgency (item 5: 2.63 vs. 2.45; *p* = 0.71) differed between groups. As noted above, the limited sample size and the distribution of stomas (only one WW patient had an ostomy, vs. eight in RS) prevented firm conclusions from being drawn about the Ostomy-related control domain. The Sexual function item showed a non-significant trend favoring the WW patient group (item 7: 2.79 vs. 2.12; *p* = 0.066).

The Psychosocial adaptation domain integrated three items: Social life (item 10) and work situation (item 11) were both significantly better in WW patients (3.76 vs. 3.06; *p* = 0.001 and 3.58 vs. 2.45; *p* = 0.0076, respectively), while family life (item 12) showed higher but non-significant scores in WW patients (3.71 vs. 3.28; *p* = 0.079).

The Mental health domain included Anxiety/worry (item 13) and perception of cure (item 14), and both showed markedly higher scores in those cases who underwent a WW management (3.71 vs. 3.33; *p* = 0.014 and 3.68 vs. 3.29; *p* = 0.016). Consistent with these findings, the overall self-rated quality of life (item 18) strongly favored WW (8.21 vs. 7.06; *p* = 0.011). Finally, the Care satisfaction domain revealed a significantly higher satisfaction with the medical team in WW patients (item 15) (3.80 vs. 3.44; *p* = 0.024), while nursing (item 16) and information provision (item 17) reported high and similar scores between the groups.

## 4. Discussion

Our analysis of the FOREST Project results adds to the growing body of evidence that organ-sparing strategies can be safely offered to patients with LARC without compromising oncological outcomes, while providing significant benefits beyond traditional endpoints. In the clinical component of this study, we observed that after completing TNT, a substantial proportion of patients achieved a sustained complete response and were managed with a WW approach. This finding is consistent with the results of the OPRA trial and other prospective series, which report that nearly half of eligible patients can avoid radical surgery after a deep response to chemoradiotherapy [21,22,23]. In our previous work [17], we showed that event-free survival was comparable between WW and RS groups, consistent with meta-analyses and registry data demonstrating non-inferior disease control for WW compared with TME [11]. Moreover, a shorter median survival was observed in the surgical subgroup, which likely reflects initial disparities: patients undergoing surgery tended to have a worse clinical condition, and all recorded deaths occurred in that group. As previous authors have cautioned, the selection bias inherent in observational designs makes it difficult to attribute survival differences to the treatment modality; randomized controlled trials remain essential to disentangle the effects of treatment from confounding factors such as tumor biology or patient frailty [10]. In this study, we performed a preliminary analysis of demographic and clinical characteristics to identify significant associations that could potentially bias the subsequent PROM comparisons. Notably, no significant associations were found with the final treatment plan for any of the analyzed parameters (Table 1).

Where this study expands upon existing literature is in the systematic capture of patient-reported outcomes alongside oncological endpoints. PROMs and PREMs are increasingly recognized as critical in modern oncology, particularly in rectal cancer, where survival is determined not only by disease control but also by functional and psychosocial sequelae [12]. Surgical morbidity, including bowel dysfunction, urgency, and incontinence, is well documented; however, broader dimensions of recovery, such as emotional well-being and social reintegration, have only recently begun to be quantified [5]. Our 18-item PROM questionnaire, coded and transformed to a 0–100 scale following the EORTC methodology, revealed clear advantages for WW patients across multiple domains. It should be acknowledged that our PROM questionnaire was adapted from validated instruments (EORTC QLQ-C30/CR29, LARS, Vaizey) but was not itself formally validated. This ad hoc approach aimed to address gaps in existing tools, particularly for patients whose condition was managed without surgery. However, we recognize this could represent a limitation and encourage future development of standardized PROMs tailored to organ-preservation strategies. Furthermore, we also considered feasibility and burden for respondents in a real-world observational cohort. It is worth noting that, despite simplification to 18 items, adherence to the questionnaire remained suboptimal, with a significant proportion of eligible patients failing to return the PROMs, and items missing from some questions. Symptoms and complications (87 vs. 66), psychosocial adjustment (90 vs. 66), and mental health (90 vs. 78) were significantly higher in WW patients. Global quality of life was also noticeably higher in WW patients (80 vs. 67; *p* = 0.011) (Table 3). These findings reinforce previous observations that organ-preserving strategies can mitigate pain, fatigue, and neuropathy, while facilitating reintegration into social and occupational roles [5,16]. Not all domains showed significant differences (bowel function and sexual health were similar across groups, and satisfaction with care was uniformly high), but the overall trend underscores the quality-of-life benefit associated with avoiding radical resection and a permanent stoma [3]. A plausible explanation for the absence of significant differences in continence and sexual function between WW and RS groups lies in the considerable heterogeneity of how patients experience and report these domains. Both groups are similarly influenced by the effects of neoadjuvant treatments, which can mask differences that are solely attributable to the therapeutic strategy. In addition, many patients lack a personal baseline or alternative reference point against which to compare their current functional status, often normalizing their symptoms as expected outcomes of rectal cancer and its treatment. This limited capacity for self-comparison likely diminishes the sensitivity of patient-reported outcomes to detect subtle discrepancies between treatment approaches. Conversely, patients who avoid surgery might have higher expectations for their functional recovery, which could influence how they perceive and rate their outcomes, further contributing to the observed similarities between groups [16,24]. Although TME is a major driver of bowel dysfunction and Low Anterior Resection Syndrome (LARS), we observed no statistically significant difference in the patient-reported “Bowel function and control” domain between the WW and RS strategies. One possible reason is that both groups were exposed to an intensive TNT backbone that included long-course chemoradiotherapy (54 Gy/30 fractions) plus systemic chemotherapy, which can itself lead to persistent anorectal dysfunction (e.g., urgency and clustering/fragmentation) even in the absence of resection. Major LARS-type symptoms have been reported in approximately one-third of WW patients after chemoradiotherapy [5], and late radiotherapy dose–effect studies within WW programs similarly identify clustering and fecal urgency as common residual complaints [25]. Radiotherapy exposure is also recognized as an independent risk factor for major LARS in surgical populations [26]. Beyond biological toxicity, PROMs may be influenced by response shift/normalization over time [27], and by expectation effects on self-assessment [28]. Finally, six patients initially assigned to the WW pathway ultimately underwent surgery but were retained in the WW arm under an intention-to-treat approach, which would be expected to attenuate observable between-group functional differences. These findings are consistent with TNT-era organ-preservation frameworks [29].

The item-level analysis (Table 4) reveals which aspects of recovery drive these differences observed between WW and RS: pain relief (items 1–2), reduced fatigue and neuropathy (items 8–9), improved social and occupational adjustment (items 10–11), and decreased anxiety (item 13) stand out as particularly influential. Conversely, the observation that some psychosocial measures (family life) and functional elements (urgency, sphincter control, sexual function) only tend to be significant highlights the heterogeneity of the patient’s experience and the need for individualized supportive care [4].

Our PROM/PREM data were collected at a single, late time point, providing a long-term snapshot rather than a longitudinal evolution. Consequently, in patients with WW, reported outcomes may incorporate the impact of local regrowth or distant relapse and subsequent rescue or systemic treatments when these occurred. Therefore, comparisons should be interpreted as reflecting the patient’s overall long-term experience with each strategy. This reflects real-world clinical practice and represents a limitation of the study. Nevertheless, restricting inclusion to patients with at least two years of follow-up allowed assessment of patient-reported outcomes beyond the acute treatment phase, when quality of life and patient experience are expected to be more stable. Furthermore, there are several other important limitations to consider. First, the sample size is modest: only 43 of 61 patients (excluding 6 cases with exitus) returned the PROM questionnaires, leaving 25 in the WW arm and 18 in the RS arm. This relatively small sample size may limit the statistical power to detect differences in some domains, particularly those with high variability or fewer respondents, such as sexual function and ostomy-related control. These findings should be interpreted with caution and validated in larger cohorts. Second, the retrospective observational design precludes causal inference and is vulnerable to unmeasured confounding; patients with a poorer baseline status were more likely to undergo surgery. Third, the follow-up period is relatively short, limiting the understanding of long-term trajectories of functional recovery and quality of life. Fourth, while our PROM instrument was based on validated scales and coded under clinical supervision, it remains personalized and not psychometrically validated, and recall and social desirability biases may influence responses. Fifth, our findings come from a single institution in Spain and may not be generalizable to other healthcare settings [30]. Finally, although baseline characteristics were similar between the groups, treatment allocation was not randomized. Clinical judgment and patient preference likely influenced decisions, and unmeasured factors, such as frailty, could have contributed to the differences in outcomes. The higher mortality in the RS group may reflect disparities in baseline health rather than the effect of the treatment. We acknowledge this potential selection bias as an inherent limitation of the study.

Beyond its scientific implications, the FOREST project exemplifies Quirónsalud’s commitment to value-based healthcare, a model that prioritizes the most important outcomes for patients. Value-based medicine demands that success be measured not only in terms of survival but also in terms of quality of life. Organ preservation after TNT is not merely a technical achievement but offers a treatment pathway that combines oncological safety with superior well-being. In fact, the inclusion of shared decision-making as a core element of the FOREST protocol may explain some deviations from standard clinical recommendations, such as patients with partial or incomplete responses opting for a watch-and-wait strategy (Table 2). This approach reflects a deliberate commitment to respecting patient autonomy and tailoring treatment beyond oncologic criteria. Importantly, shared decision-making in FOREST is systematically grounded in a multidimensional evaluation of each patient’s quality of life and care experience—dimensions that are explicitly captured through PROMs and PREMs. By integrating these patient-reported measures into the clinical dialogue, the protocol ensures that therapeutic decisions are informed not only by tumor response but also by the patient’s values, expectations, and lived experience.

## 5. Conclusions

In summary, these results contribute to the growing movement on personalized, patient-centered cancer care and reinforce the principle that high-value rectal cancer treatment must combine oncological rigor with empathy, engagement, and attention to quality of life [31]. Moreover, our findings here are consistent with the latest results from the FOREST protocol [18], which confirmed that a response-adapted WW strategy not only maintains oncologic safety but also improves overall survival compared to radical surgery. This evidence strengthens the value of individualized, organ-preserving approaches to rectal cancer management. Future research should validate these findings in larger prospective cohorts, extend follow-up to capture long-term outcomes, and refine PROM tools for broader applicability. These efforts will ensure that organ preservation strategies evolve from promising innovations to enduring components of value-based clinical practice.

## Figures and Tables

**Table 1:** Association of clinical and molecular characteristics with the final therapeutic plan in our study cohort of 67 LARC patients.

	No. Cases	No. Watch and Wait (%)		No. Radical Surgery (%)		*p*
Therapeutic Plan	43	25 (58.1)		18 (41.9)		
Gender	43	25		18		0.640
Male	28	17	(60.7)	11	(39.3)	
Female	15	8	(53.3)	7	(46.7)	
Age	43	25		18		0.931
<60	15	8	(53.3)	7	(46.7)	
≥60	28	17	(60.7)	11	(39.3)	
Histological subtype	38	22		16		0.816
Conventional	36	21	(58.3)	15	(41.7)	
Mucinous	2	1	(50)	1	(50)	
Late morbidity	38	21		17		0.258
No	28	17	(60.7)	11	(39.3)	
Yes	10	4	(40)	6	(60)	
Relapse	42	25		17		0.439
No	32	18	(56.3)	14	(43.7)	
Local and/or distant	10	7	(70)	3	(30)	
Patient Status	43	25		18		0.953
Alive without Disease	36	21	(58.3)	15	(41.7)	
Alive with Disease	7	4	(57.1)	3	(42.9)	

**Table 2:** Final therapeutic decision and outcomes according to early evaluation (week 4–6) and late evaluation (week 20–26) results.

	No. Cases	No. Watch and Wait (%)		No. Radical Surgery (%)		*p*
Therapeutic Plan	43	25 (58.1)		18 (41.9)		
Early Evaluation	43	25		18		0.104
End TNT ^1^	6	6	(100)	0	(0)	
Complete Standard TNT regimen (6 cycles)	28	15	(53.6)	13	(46.4)	
Complete Extended TNT regimen (8 cycles)	48	4	(50)	4	(50)	
Direct Radical Surgery	1	0	(0)	1	(100)	
Late Evaluation	39	32		26		<0.001
Complete Response	16	16	(100)	0	(0)	
Almost CR ^2^	11	5	(45.5)	6	(34.5)	
Incomplete Response	12	1	(8.3)	11	(91.7)	

^1^ TNT: total neoadjuvant therapy; ^2^ CR: complete response.

**Table 3:** Domain-level analysis of PROM questionnaires.

	Watch and Wait		Radical Surgery		
Domain	Mean	SD	Mean	SD	*p*
Symptoms and complications	87.5	9.1	62.5	16.7	<0.001
Bowel function and control	62.5	36.9	56.1	43.0	0.703
Ostomy-related control	100	-	79.2	35.4	0.643
Sexual function	59.7	40.5	37.5	34.2	0.066
Psychosocial adaptation	90.4	15.3	65.7	24.3	<0.001
Mental health	90.0	13.6	77.8	12.8	0.006
Care satisfaction	93.3	10.6	86.4	14.0	0.105
Global quality of life	80.1	16.7	67.3	16.4	0.011

**Table 4:** Item-level analysis of PROM questionnaires.

	Watch and Wait			Radical Surgery			
Item	N	Mean	SD	N	Mean	SD	*p*
1. Regarding the symptoms I had when I started the process before treatment	25	3.92	0.28	18	3.56	0.51	0.006
2. Regarding the pain after my treatment	25	3.88	0.44	16	3.44	0.81	0.024
3. Regarding the complications or sequelae of my treatment	25	2.88	0.97	18	2.28	1.02	0.056
4. Regarding my sphincter control	22	3.18	1.30	10	3.10	1.45	0.980
5. Regarding the need to rush to the bathroom	24	2.62	1.24	11	2.45	1.21	0.712
6. Regarding the ostomy bag	1	4.00	-	8	3.38	1.06	0.643
7. My sex life	24	2.79	1.22	16	2.12	1.02	0.066
8. Regarding tiredness	25	3.92	0.28	18	3.28	0.46	<0.001
9. Regarding tingling and/or numbness in the feet and hands	25	3.52	0.87	17	2.53	0.80	<0.001
10. I live my social life normally, or as I would like	25	3.76	0.60	18	3.06	0.80	0.001
11. Regarding my work situation	19	3.58	0.96	11	2.45	1.29	0.008
12. Regarding my relationship with my family, the outcome of my treatment has not affected my pace of life	24	3.71	0.55	18	3.28	0.89	0.079
13. Worry or anxiety	24	3.71	0.55	18	3.33	0.49	0.014
14. I feel that I have healed	25	3.68	0.48	17	3.29	0.47	0.016
15. My satisfaction with the medical team	25	3.80	0.50	18	3.44	0.62	0.024
16. My satisfaction with the nursing team	25	3.92	0.28	18	3.78	0.43	0.196
17. My satisfaction with the information received	25	3.68	0.48	18	3.56	0.51	0.419
18. On a scale of 1 to 10, how I would rate my quality of life	24	8.21	1.50	18	7.06	1.47	0.011

## Data Availability

The data underlying this article are available in the article and in its online Supplementary Material.

## Supplementary Materials

The following supporting information can be downloaded at https://www.mdpi.com/article/10.3390/jcm15020844/s1, Table S1: Clinical and demographic features characteristics of the LARC patient cohort; Table S2: Ordinal coding scheme for each item of the PROMs questionnaire; Table S3: Domain-level analysis of PROMs: items by domain.
