# Correction: Guadalajara et al. Impact on Clinical- and Patient-Reported Outcomes Measures of an Organ Preservation-Based Therapeutic Strategy in Locally Advanced Rectal Cancer: The FOREST Project. *J. Clin. Med.* 2026, *15*, 844

**DOI:** 10.3390/jcm15155883

**Published:** 2026-07-28

**Authors:** Hector Guadalajara, Ion Cristóbal, Raquel Fuentes-Mateos, Eva Ruiz-Hispán, Jose Luis Domínguez-Tristancho, Miguel Leon-Arellano, Paula Sánchez-Moreno, Marta Sabater-Durán, Juan Antonio Álvaro de la Parra, Damián García-Olmo, Cristina Caramés

**Affiliations:** 1 Department of General and Digestive Surgery, Fundación Jiménez Díaz University Hospital, 28040 Madrid, Spain; h.guadalajara@quironsalud.es (H.G.); jluis.dominguezt@quironsalud.es (J.L.D.-T.); miguel.leon@quironsalud.es (M.L.-A.); damian.garcia@quironsalud.es (D.G.-O.); 2 Department of Surgery, School of Medicine, Autonomous University of Madrid, 28049 Madrid, Spain; 3 Corporate Department of Healthcare and Research, Quirónsalud Healthcare Network, 28043 Madrid, Spain; psmo.edu@quironsalud.es; 4 Medical Oncology Department, Fundación Jiménez Díaz University Hospital, 28040 Madrid, Spain; raquel.fmateos@quironsalud.es (R.F.-M.); eva.ruizh@quironsalud.es (E.R.-H.); 5 Project Implementation and Processes Department, Quirónsalud Healthcare Network, 28043 Madrid, Spain; marta.sabaterd@quironsalud.es; 6 General Operations Directorate, Quirónsalud Healthcare Network, 28043 Madrid, Spain; jaalvaro@quironsalud.es; 7 Doctoral School of the UAM, Center for Postgraduate Studies, Autonomous University of Madrid, 28049 Madrid, Spain; 8 Doctoral Program in Medicine and Surgery, Autonomous University of Madrid, 28049 Madrid, Spain

## Additional Affiliations

In published publication [1] there was an error regarding the affiliations for Juan Antonio Álvaro de la Parra, Damián García-Olmo and Cristina Caramés. In addition to original affiliations, the updated affiliations should include “Doctoral School of the UAM, Center for Postgraduate Studies, Autonomous University of Madrid, 28049 Madrid, Spain” for Juan Antonio Álvaro de la Parra and “Doctoral Program in Medicine and Surgery, Autonomous University of Madrid, 28049 Madrid, Spain” for Damián García-Olmo and Cristina Caramés. The authors state that the scientific conclusions are unaffected. This correction was approved by the Academic Editor. The original publication has also been updated.
