# Supplementary material for: Impact on Clinical- and Patient-Reported Outcomes Measures of an Organ Preservation-Based Therapeutic Strategy in Locally Advanced Rectal Cancer: The FOREST Project
Source: J Clin Med. 2026 Jan 20;15(2):844. doi: 10.3390/jcm15020844 (PMC12842394; doi:10.3390/jcm15020844)
Supplement: Supplementary file 1 [file jcm-15-00844-s001.zip › Table S1.pdf]

Table S1. Clinical and demographic features characteristics of the LARC patient cohort.

		No.	(%)
Gender			
	Male	28	(65.1)
	Female	15	(34.9)
Age			
	<60	15	(34.9)
	≥60	28	(65.1)
Histological subtype			
	Conventional	36	(94.7)
	Mucinous	2	(5.3)
Late morbidity			
	No	28	(73.7)
	Yes	10	(26.3)
Relapse			
	No	32	(76.2)
	Local and/or distant	10	(23.8)
Patient Status			
	Alive without Disease	36	(83.7)
	Alive with Disease	7	(16.3)
