# Supplementary material for: Impact on Clinical- and Patient-Reported Outcomes Measures of an Organ Preservation-Based Therapeutic Strategy in Locally Advanced Rectal Cancer: The FOREST Project
Source: J Clin Med. 2026 Jan 20;15(2):844. doi: 10.3390/jcm15020844 (PMC12842394; doi:10.3390/jcm15020844)
Supplement: Supplementary file 1 [file jcm-15-00844-s001.zip › Table S2.pdf]

Table S2. Ordinal coding scheme for each item of the PROMs questionnaire.

1. Regarding the symptoms I had when I started the process before treatment:	
**Option**	**Code**
I am worse	1
I have not improved	2
I have improved a little	3
I have resolved the symptoms	4
2. Regarding the pain after my treatment:	
**Option**	**Code**
I have a lot of pain and need high doses of analgesics	1
I have some pain and with some analgesic it is enough	2
The pain is bearable without analgesia	3
I have no pain	4
3. Regarding the complications or sequelae of my treatment:	
I have suffered many complications and I have significant sequelae remaining	1
I have suffered some complication with few sequelae	2
I have suffered some complication with no sequelae	3
I have not suffered complications	4
4. Regarding my sphincter control:	
I am an ostomy carrier	N/A
I need to wear a pad always	1
I wet myself once a day	2
I wet myself once a week	3
I wet myself very little or never	4

5. Regarding the need to rush to the bathroom:	
**Option**	**Code**
I am an ostomy carrier	N/A
Daily	1
Several times a week	2
Less than once a week	3
Almost never or never	4
6. Regarding the ostomy bag:	
**Option**	**Code**
I do not have an ostomy bag	N/A
Someone has to take care of my stoma	1
I can take care of my stoma, but I need constant help	2
I can take care of my stoma with occasional help	3
I can take care of my stoma without needing help	4
7. My sex life:	
I cannot have sexual relations	1
My sexual relations are usually painful or unsatisfactory	2
My sexual relations are unsatisfactory occasionally	3
My sex life is satisfactory or has not changed	4
8. Regarding tiredness:	
I have no strength to get out of bed	1
I have no strength to leave the house	2
Fatigue allows me to leave the house and do basic activities, but with limitations	3
Fatigue is occasional or I do not experience fatigue, so I can live a life similar to before treatment	4

9. Regarding tingling and/or numbness in the feet and hands:	
**Option**	**Code**
It is disabling; it prevents me from walking or dressing	1
The discomfort is continuous, but it allows me to perform basic daily tasks	2
Sometimes I have numbness	3
It is very occasional, or I do not have it	4
10. I live my social life normally, or as I would like:	
**Option**	**Code**
No, in no aspect and it is very limiting	1
I cannot adapt in all aspects, but my life is functional	2
Yes, I have managed to adapt with great effort	3
Yes, I have managed to adapt with relative ease	4
11. Regarding my work situation:	
**Option**	**Code**
I have not been able to start any job	1
I have had to change my job	2
I have had to adapt my job position	3
I have been able to do my job normally again	4
12. Regarding my relationship with my family, the outcome of my treatment has not affected my pace of life:	
**Option**	**Code**
We have broken family ties	1
The difficulties have caused a family estrangement	2
We have overcome the problems with difficulty without impacting our relationship	3
It has had practically no repercussions on my family life	4

13. Worry or anxiety:	
**Option**	**Code**
It is all I think about constantly	1
I feel worried and anxious most of the day	2
It worries me but I live with it	3
It affects me occasionally, or it does not affect me at all	4
14. I feel that I have healed:	
**Option**	**Code**
I am the same or worse, I do not feel cured	1
I have improved very little, but I still feel sick	2
I have improved a lot, but I do not feel completely cured	3
I feel cured	4
15. My satisfaction with the medical team:	
**Option**	**Code**
Very low	1
Low	2
High	3
Very high	4
16. My satisfaction with the nursing team is:	
**Option**	**Code**
Very low	1
Low	2
High	3
Very high	4

17. My satisfaction with the information received is:	
**Option**	**Code**
Very low	1
Low	2
High	3
Very high	4
18. On a scale of 1 to 10, how I would rate my quality of life:	
**Option**	**Code**
Score 1 to 10	N/A
Patients´ comments	
**Option**	**Code**
Free text	N/A
