# Supplementary material for: Impact on Clinical- and Patient-Reported Outcomes Measures of an Organ Preservation-Based Therapeutic Strategy in Locally Advanced Rectal Cancer: The FOREST Project
Source: J Clin Med. 2026 Jan 20;15(2):844. doi: 10.3390/jcm15020844 (PMC12842394; doi:10.3390/jcm15020844)
Supplement: Supplementary file 1 [file jcm-15-00844-s001.zip › Table S3.pdf]

Table S3. Domain-level analysis of PROMs: items by domain.

Domain	Items included
Symptoms and complications	1 (symptoms), 2 (pain), 3 (treatment complications/sequelae), 8 (fatigue), 9 (neuropathy)
Bowel function and control	4 (sphincter control), 5 (urgency), noting that these items do not apply to stoma patients
Ostomy-related control	6 (ostomy bag care)
Sexual function	7 (sexual life)
Psychosocial adaptation	10 (social life), 11 (work situation), 12 (family life)
Mental health	13 (anxiety/worry), 14 (perception of being cured)
Care satisfaction	15 (satisfaction with medical team), 16 (with nursing team), 17 (with information provided)
Global quality of life	18 (overall QoL score)
